# Shear Bond Strength and Fluoride Release of a Universal Adhesive: An In-Vitro Study on Primary Teeth

**DOI:** 10.3390/ma16072573

**Published:** 2023-03-23

**Authors:** Alaa Alsaadawi, Osama Felemban, Hani M. Nassar, Medhat Abdelbaki

**Affiliations:** 1 Department of Pediatric Dentistry, King Abdulaziz University, Jeddah 21589, Saudi Arabia; 2 Department of Restorative Dentistry, King Abdulaziz University, Jeddah 21589, Saudi Arabia

**Keywords:** Clearfil UBQ, fluoride release, shear bond strength, primary teeth, universal adhesive, Scotchbond, glass ionomer

## Abstract

This investigation aimed to assess the shear bond strength and fluoride-releasing capabilities of Clearfil Universal Bond Quick (Kuraray Noritake Dental Inc., Tokyo, Japan). Forty-four extracted primary molars were divided into two groups, and the enamel substrate was prepared for evaluating shear bond strength. Scotchbond (3M ESPE) and Clearfil UBQ were used to bond composite-to-enamel substrates in each group (*n* = 22). Shear bond strength was measured using a universal testing device and compared. Sixteen discs (6 mm diameter and 3 mm thickness) were fabricated from each Clearfil UBQ, Fuji IX, and Fuji II LC. Over the course of 30 days, each materials’ fluoride release was examined and compared using ion analysis. Results revealed that Clearfil UBQ had statistically similar shear bond strength to Scotchbond. Between the three materials, Clearfil UBQ had the lowest fluoride release at baseline (0.11 ± 0.25) and the lowest cumulative fluoride release (0.12–0.27 ppm) over 30 days. Fuji IX had the highest fluoride release at baseline (19.38 ± 2.50) and cumulatively (40.87 ± 4.03 ppm), followed by Fuji II LC. We conclude that Clearfil UBQ and Scotchbond showed comparable bond strengths to the enamel. Fluoride release was seen in Clearfil UBQ in the initial two days of the 30-day period. The amount of fluoride release was significantly less than with glass ionomer cements.

## 1. Introduction

Dental caries, more commonly known as “tooth decay”, is the most frequent oral disease around the world. Estimates suggest that over half a billion children aged 2–11 years suffer from dental caries every year and require restorations [1,2]. The growing emphasis on aesthetic considerations and a preference for minimally invasive cavity preparation techniques have led to an increase in demand for resin composites [3,4]. Considerable research into the field of dental materials has led to innovations and the adoption of innovative adhesive restorative materials.

An ideal adhesive used in the oral cavity must withstand many insults—prolonged or permanent immersion in liquids and extreme humidity. It must resist pH changes, temperature, and stress fluctuations, all the while remaining stable. The success of a restoration depends on a strong and long-lasting bond between restoration and substrate. The quality of a bond is governed by numerous factors ranging from the bonding mechanism, the specific surface treatment of the substrate, and even the mechanism used to prepare the cavity. Shear bond strength testing is a well-recognized method to evaluate the bonding performance of an adhesive to assess the strength of the bond between a dental restoration and the tooth structure. Bonding tests conducted independently in a laboratory can, to an extent, be a predictor of clinical performance.

Bonding agents that help adhere the composite to the substrate can be applied in two main modes: etch-and-rinse (ER) and self-etch (SE) modes. Etch-and-rinse refers to the traditional method of first applying an etchant on the tooth surface, after which the etched surface is rinsed then treated with a bonding agent. The self-etch mode serves to simplify the bonding procedure by using a single agent that etches and primes the tooth surface without rinsing. Universal or multimode adhesives are innovative bonding agents that give the clinicians procedural freedom, and allow the operator to choose the mode of application based on the clinical situation [5]. Universal adhesives can be used to bond to a variety of substrates enamel, dentin, resin, ceramic, zirconia, and alloys [6].

Fluoride-releasing adhesives have been identified as a potential solution to address the issue of demineralization and weakening of restorations that can occur over time as a result of pH changes and bacterial activity. A fluoride-releasing adhesive can have protective effects on the dentin and enamel around a restoration [7]. Fluoride ions are incorporated into the crystal structure of the enamel, making it more resistant to acid attacks from bacteria. Adhesives that release fluoride ions protect dental hard tissues against demineralization and acid neutralization, which is typically a part of the carious process.

Fluoride-containing bonding agents are more durable and superior to non-fluoridated bonding agents, as fluoride can improve the bond’s longevity and quality [8]. Fluoride-containing adhesives are able to form an acid-resistant zone that can limit the action of acids in case of secondary caries. Fluoride released from an adhesive could prevent degradation of the hybrid layer by reducing the solubility of calcium phosphate. This could serve to stabilize bond strength [9]. Fluoride also helps to remineralize areas of the enamel that have been damaged by acid, repairing small cavities before they become larger. Unlike inert adhesives, fluoride-releasing adhesives have anticariogenic and antibacterial properties, inhibiting the growth and activity of bacteria that cause cavities [10]. Therefore, integrating fluoride into bonding agents has the potential to minimize microleakage by effectively adhering to the tooth structure, thereby minimizing the development of secondary caries [11,12]. Previous studies have shown that glass ionomer cements (GICs) and resin-modified glass ionomer cements (RM-GICs) release fluoride over an extended period [13,14]. This fluoride release can lead to significant reductions in enamel and dentin demineralization as opposed to non-fluoridating restoratives [15,16,17].

Clearfil Universal Bond Quick (Clearfil UBQ, Kuraray Noritake Dental Inc. Tokyo, Japan) is a fluoride-containing, universal bonding agent that can be used with or without a separate etching procedure. It consists of a single bottle of primer/bonding agent. When applied as an etch and rinse to etch the enamel, it creates a roughened surface for micromechanical retention of the bonding agent. The manufacturer asserts that this product produces a speedy bond due to its ease of use and three-second application time [18]. The reduced technique sensitivity and versatility of this product may be advantageous in pediatric dentistry when dealing with younger patients, as it minimizes chair time and clinical steps [19].

Previous research has primarily focused on evaluating the shear bond strength of adhesives to the enamel of permanent teeth rather than primary teeth [20,21,22]. The literature on bonding adhesives for primary teeth lacks sufficient detail regarding the shear bond strength that can be achieved in this type of dental tissue [23,24]. The bond strength of universal adhesives to primary enamel is not well understood, and the data on fluoride release from these adhesives is limited. The current study aimed to assess the shear bond strength and fluoride-releasing capabilities of Clearfil UBQ on primary enamel. The null hypothesis of the study is that there is no significant difference between the shear bond strength of Clearfil UBQ and Scotchbond™ Universal bond, or the fluoride release from Clearfil UBQ and glass ionomer cements.

## 2. Materials and Methods

### 2.1. Study Design

The study protocol was approved by the Institutional Review Board and ethical committee of King Abdulaziz University in Jeddah, Saudi Arabia. The research was divided into two experiments, each focusing on a distinct component of Clearfil UBQ. The first experiment compared Clearfil UBQs shear bond strength to Scotchbond™ on enamel of primary teeth. The second test compared Clearfil UBQs fluoride-releasing performance to that of conventional GIC (Fuji IX) and a Resin Modified Glass Ionomer (RMGI), Fuji II LC.

### 2.2. Shear Bond Strength Experiment

For testing the shear bond strength of Clearfil UBQ to enamel, we collected 44 non-carious human primary molars with the individual’s informed consent. Teeth having cracks, fractures, or developmental anomalies were not included. A sample size calculation was performed. The expected mean difference in the shear bond strength would be approximately 2.8 MPa and the standard deviation to be 2.4 Mpa. Twenty-two samples per group were required to detect a statistically significant difference in shear bond strength between the two groups at the level of significance of 0.05 with a power of 95%. The teeth were mechanically cleaned with pumice and rubber prophylactic cups to eliminate any debris, before being preserved in a 0.5% Chloramine T and distilled water solution (Sigma Chemical Company, St. Louis, MO, USA). The 44 primary molar crowns were sectioned 1 mm apical to the CEJ. The crowns were detached from the roots with the aid of a KG Sorensen diamond double-faced disk (Barueri, SP, Brazil) under water spray. The crowns were embedded in self-curing acrylic cylindrical resin blocks measuring 20 mm in diameter and 10 mm in height with the buccal surface of the tooth exposed. The buccal surfaces of the teeth were polished with silicon carbide paper under water cooling to achieve a flat enamel surface.

Twenty-two enamel surfaces were etched and rinsed using Clearfil UBQ as per the manufacturer’s instructions. Thirty-five percent phosphoric acid was used to etch the enamel surfaces for 10 s, and the etched surface was rinsed and dried until the surfaces of the etched enamel appeared frosty white. Thereafter, Clearfil UBQ was applied with a rubbing motion and it was dried with low-pressure air for five seconds. OrtholuxTMLuminous blue LED Curing Light (3M Unitek, Monrovia, CA, USA) with a MW/cm^2^ output of 1600 was used to cure the adhesive for 10 s at a distance of 1 mm at 90 degrees.

Etch-and-rinse bonding agent Scotchbond™ Universal Bond (3M ESPE, Seefeld, Germany) was applied on the remaining twenty-two enamel surfaces as recommended by the manufacturer. For 15 s, the enamel was etched with 32% phosphoric acid, then rinsed and dried with gentle air pressure. Once the adhesive had been applied, a gentle stream of air was used for about five seconds before the adhesive was applied and light-cured for ten seconds using the OrtholuxTM Luminous blue LED Curing Light (3M Unitek, Monrovia, CA, USA) with a MW/cm^2^ output of 1600 at a distance of 1 mm at a 90-degree angle.

**Figure 1:**
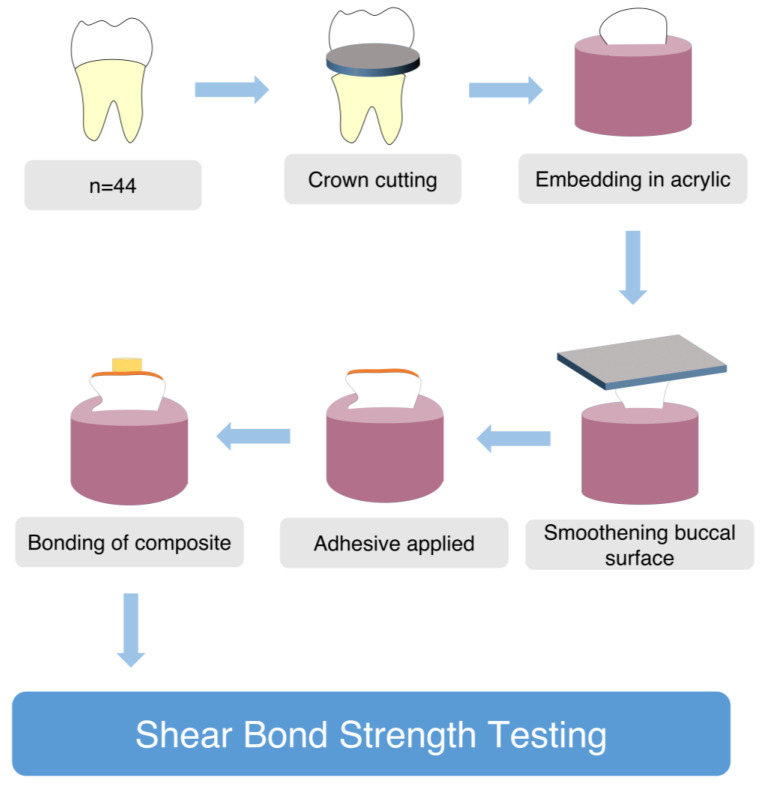
Preparation of specimens for Shear Bond Strength testing.

A shade A1 composite (Z250, 3M, ESPE, USA) was packed into a 5 mm diameter and 2 mm high cylindrical plastic mold while the mold was positioned on each of the prepared surfaces of the tooth specimens. For 20 s, the composite was held in situ and cured at a distance of one millimeter at a 90 degree angle. After separating the mold from the composite, it was further cured for 40 s. A scalpel was used to remove any superfluous material. Samples were kept at room temperature for 24 h in distilled water (Figure 1).

### 2.3. Testing Shear Bond Strength

A universal testing machine was used to measure the shear bond strength (Instron 5944, Instron Corporation, Canton, MA, USA). At a crosshead speed of one millimeter per second, specimens were subjected to a compressive force on the contact between the tooth/composite assembly. The force at which the specimen debonded was the shear force at fracture.

### 2.4. Fluoride Release

Data from a previous study on fluoride release were used to estimate the sample size for the fluoride release data study [25]. Assuming that the fluoride release of Clearfil Universal Bond Quick is 18 ppm after 30 days and using an effect size of 1.2, it was determined that 16 samples per group were necessary for the study to detect statistical significance between the groups in fluoride release with a 95 percent power at the significance level of 0.05.

### 2.5. Sample Preparation

For testing the fluoride release of Clearfil UBQ, we created sample discs of the material along with discs made of glass ionomer cements. All discs were created following the manufacturer’s directions for material handling. All specimens were made by a single operator. GIC capsules of Fuji II LC (GC Corp. Tokyo, Japan) and Fuji IX GP (GC Corp, Tokyo, Japan) were activated and mixed for 10 s in an amalgamator at room temperature. Sixteen samples of each material were placed in a total of 48 cylindrical acrylic mold. Clearfil UBQ and the Fuji II LC were cured using an OrtholuxTM Luminous Curing Light (3M Unitek, Monrovia, CA, USA) The Fuji IX GP was allowed to self-cure for 3 min. All samples were removed from the mold, resulting in a disc-shaped sample 6 mm in diameter and 3 mm thick.

### 2.6. Fluoride Release Testing

Individual disc-shaped specimens were immersed in fluoride-free polypropylene vials containing 5 mL of deionized water and placed in a 37 °C incubator for 24 h. Every day for the first five days, the discs were rinsed and put into a new plastic vial containing fresh deionized water. The specimens were then rinsed and put into new vials every five days for the next 30 days. We pipetted 1 mL of the storage media from each day into a fresh vial and combined it with 1 mL of TISAB II solution (Total Ionic Strength Adjustment Buffer; perfection; Mettler Toledo, Greifensee, Switzerland) to prepare the specimens for fluoride release measurement. The concentration of fluoride released was determined using a fluoride electrode (Orion Research, Inc., Boston, MA, USA) linked to an ion analyzer (Model 214A, Orion Research).

### 2.7. Statistical Analysis

Means and standard deviation were used to describe quantitative variables using descriptive statistics. The Shapiro-Wilk Test was used to determine whether the shear bond strength and the fluoride release data were normally distributed. The shear bond strength data were found to be normally distributed. Hence, an independent sample t-test was applied for the two-group comparison. The normality of the fluoride release and homogeneity of the variances was found to be not normally distributed. Therefore, an independent sample Kruskal-Wallis test followed by post hoc analysis was used for the fluoride release. Data obtained through experiments were entered into separate spreadsheets and analyzed using IBM SPSS Statistics for Windows, Version 23.0 (SPSS Inc., Chicago, IL, USA).

## 3. Results

### 3.1. Shear Bond Strength

The mean shear bond strength for Clearfil UBQ was determined to be 15.82 ± 0.88 MPa, while the mean shear bond strength for Scotchbond was recorded as 16.25 ± 0.73 MPa. Results of the independent sample *t*-test revealed that there was no statistically significant difference in the bond strengths between Clearfil UBQ and Scotchbond (*p* = 0.54). Regardless of the adhesive used, both Clearfil UBQ and Scotchbond exhibited similar bond strengths when used on enamel. The outcomes of the shear bond strength testing are presented in Table 1.

### 3.2. Fluoride Release

The fluoride release patterns varied between the three materials at all time points. From baseline to day 30, there were differences in fluoride release across the three groups that were statistically significant. The various fluoride release patterns of the three materials are depicted in Table 2.

**Figure 2:**
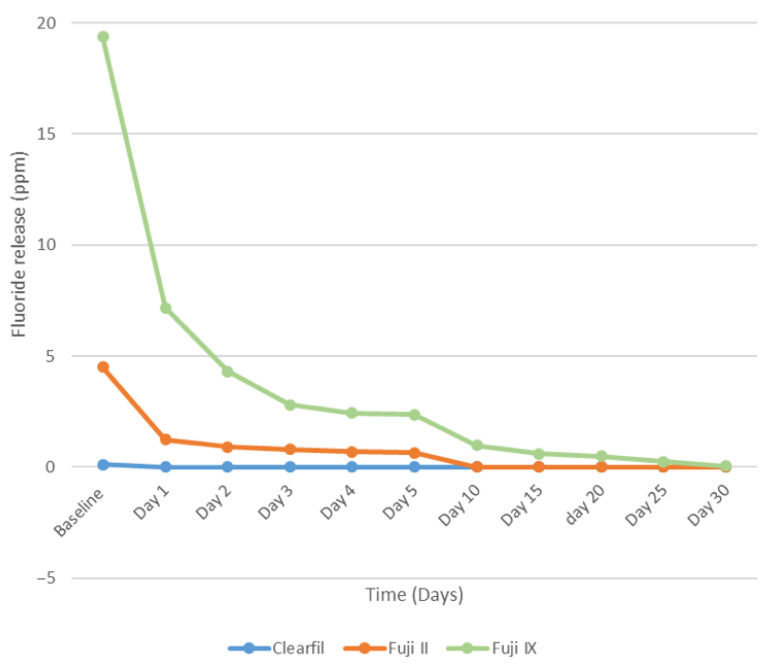
Comparison of Fluoride released from the three materials at each time point for 30 days.

The statistical analysis at Baseline, Day 1, Day 2, Day 3, Day 4, and Day 5 revealed a significant difference among the groups (*p* < 0.001). Clearfil UBQ and Fuji II both exhibited a similar fluoride release from Day 10 to Day 30. However, from Day 10 to Day 30, Fuji IX demonstrated a statistically significant higher fluoride release in comparison to Clearfil UBQ and Fuji II (*p* < 0.001). Figure 2 shows that the greatest amount of fluoride release took place in the first 24 h at baseline for all three materials. A sharp decline was seen on the following day.

**Figure 3:**
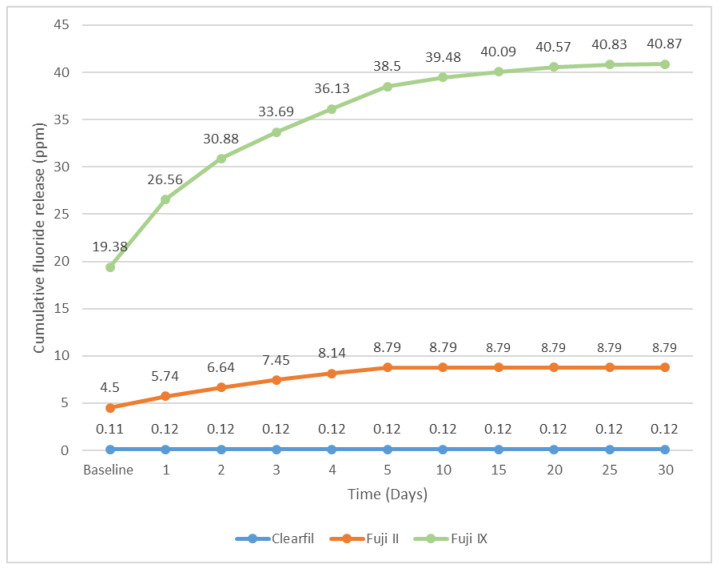
Comparison of cumulative Fluoride release over 30 days among the three materials.

The data presented in Figure 3 indicates a significant correlation between the type of material and the cumulative fluoride concentration in the medium. The ranking of materials in terms of fluoride release is as follows: Fuji IX > Fuji II > Clearfil UBQ. Glass ionomer cements demonstrated prolonged fluoride release over the duration of the study. Among the glass ionomer cements examined, the resin-modified GIC (Fuji II LC) exhibited a lower rate of fluoride release in comparison to the conventional GIC (Fuji IX).

## 4. Discussion

Measuring the shear bond strength of an adhesive is a widely accepted method for assessing bond fracture resistance, which is a key factor in determining the long-term durability of restorations. In this study, we examined the bond strength and fluoride release of Clearfil UBQ [26].

Our findings indicate that Clearfil UBQ, when used in an etch-and-rinse mode, provides an effective adhesive strength to primary enamel. The shear bond strength of Clearfil UBQ to enamel closely matched that of the control used i.e., Scotchbond. The null hypothesis was not rejected.

Our findings align with earlier observations by Sato et al., who reported that Clearfil UBQ had excellent shear bond strength comparable to G-Premio BOND (GPB, GC, Tokyo, Japan) [7]. Clearfil UBQ contains a new hydrophilic amide monomer that can easily penetrate the enamel substrate, establishing a chemical and mechanical adhesion to the tooth surface [27]. Our results reflect the findings of Papadogiannis et al., who reported that Clearfil UBQ had a bond strength that was greater than G-Premio BOND and comparable to Scotchbond [28]. Consistent with the literature, our findings broadly support the work of other studies in this area, confirming the adhesive capacity of Clearfil UBQ.

Earlier studies have found that fluoride-releasing adhesives provide sufficient bond strength [29,30,31]. Literature does not reveal an exact optimal bond strength for universal adhesives. Previous reports of universal adhesives’ bond strengths range from 5.53 to 22 [32,33]. The bond strengths of Scotchbond to enamel achieved in our study fall within this range and outstrip previous reports (16.25 Mpa). Hellak et al. reported a much lower enamel bond strength (12.06 MPa) with Scotchbond [34]. This difference in bond strength may be attributed to the difference in methodology. Hellak et al. used a self-etch mode that may have contributed to lower bond strength, while our investigation employed an etch and rinse mode. Etch-and-rinse and self-etching modes produce different etching patterns. Phosphoric acid (37%) etching causes large pits in the hydroxyapatite-rich enamel, allowing the resin to penetrate the enamel more deeply and so resulting in a stronger bonding [35]. In the self–etching bonding mode, the smear layer is incorporated into the bonding substrate with shortened resin tags, which could contribute to decreased bonding strengths [36].

It is important to note that the results of shear bond strength testing may be influenced by a variety of factors, including the type of adhesive, the surface preparation of the tooth, and the type of dental restoration being used. Therefore, it is important to use standardized testing protocols and the specific conditions under which the test was performed. Differences in testing substrate could have an effect on shear bond strength. Nagura et al. observed an enamel bond strength of 20.9 MPa with Scotchbond, which differs considerably from our result [37]. This difference in the bond strengths can be attributed to the different substrates used—unlike Nagura et al., who used permanent teeth. Adhesive strength is often lower in primary teeth compared to permanent teeth as they are fundamentally different in composition and form [38]. Thinner primary enamel, lower density, different direction and number of enamel prisms are some of the examples of the difference between primary and permanent teeth [39]. While a high shear bond strength may indicate good bonding performance in laboratory testing, it may not necessarily translate to good clinical performance. Therefore, it is crucial to combine the laboratory results with clinical evaluations to gain a comprehensive understanding of the adhesive performance.

We examined the fluoride-releasing capacity of Clearfil UBQ over 30 days and compared it to conventional and resin-modified glass ionomer cements (RMGIC). The results showed that the three materials differed significantly in the fluoride release patterns leading to a rejection of the null hypothesis.

Among the restorative materials, the glass ionomers had greater fluoride release than the universal adhesive. The highest fluoride release was seen in Fuji IX, followed by Fuji II (RMGIC). This finding is consistent with previous research [40,41,42]. The high fluoride content and increased water uptake by Fuji IX is presumably the reason for its higher fluoride release. Clearfil UBQ had the least amount of fluoride release.

The difference in fluoride released is likely due to the setting mechanism involved. GICs release fluoride from fluoridated alumino-silicate glass, which dissolves in the solution. Fluoride-releasing Composite resins act through ion exchange from the filler to the solution. Fluoride is released in smaller quantities due to an insufficient availability of H^+^ ions in a tightly polymerized hydrophobic resin [43].

All three materials released the most fluoride in the first 24 h. The initial high levels of fluoride release can be ascribed to the “burst phenomenon” observed in the setting of GICs. The initial burst of high fluoride release is due to the acid-base setting reaction of the GIC [44]. Fluoride release declines rapidly during the first week, going on to stabilize after 3–4 weeks. The burst phenomenon of fluoride release may have beneficial biological effects. It is postulated to have a bactericidal effect immediately after restoration [45].

Fluoride release is a complicated process. The solubility, permeability, and porosity of the mass, as well as its surface area (for treatment and finishing), as well as other internal and external factors, including organic matrix and filler composition, pH of the material, and manipulation method, all have a role. As a result of various experimental techniques, published data on fluoride release varies widely. Fluoride release values generated during in-vitro testing have been shown to be higher than during in-vivo testing, according to previous investigations [40,46,47]. As in our experiment, this could possibly be due to the increased surface area of the adhesive disc used to examine fluoride release. Our findings of an initial burst release of fluoride ions followed by tapering off at later time points are broadly supported by and in agreement with previous reports from Pellizari et al. and May and Donly [48,49].

Fluoride ion release serves to promote mineralization and decelerate demineralization, contributing to the longevity of the restoration [7,50]. Adhesives that release fluoride have a cariostatic effect, inhibiting the formation of new cavities underneath the restoration. Fluoride-containing adhesives can create an acid-resistant zone to minimize the risk of secondary caries. Fluoride can prevent enzymes that break down collagen and ester bonds from degrading the hybrid layer by inhibiting their activity [51]. The solubility of calcium phosphate could be reduced by fluoride produced from adhesives, which could delay the deterioration of the hybrid layer. Increasing the soluble stability of calcium phosphate may help maintain the strength of the bond [9].

In contrast to GICs, the fluoride release from Clearfil UBQ was much lower despite its claim to be a fluoride-releasing adhesive. This may be due to its distinct composition, filler size, kind of fluoride filling particles, and resin type [52]. The sodium fluoride filler in Clearfil UBQ is water-soluble. This filler particle’s solubility helps the adhesive’s fluoride release [8]. The exact amount of fluoride required to prevent secondary caries remains a point of contention [53]. Fluoride concentrations below 1 ppm have been shown to minimize demineralization and enhance remineralization [54]. Lapinksa et al. reported that Clearfil UBQs release sufficient fluoride to exert antibacterial properties, disrupting the physical membrane of *S. mutans* [55].

This in vitro study adds data to the evidence base for Clearfil UBQ universal adhesives’ enamel bond strength and fluoride release. Although in vitro studies can provide data that can be extrapolated to clinical practice, several factors may hinder an accurate simulation of intraoral conditions. Future studies can consider bond strength as a function of time. Testing shear bond strength after artificially aging the specimens and thermocycling post-restoration could provide a glimpse into bond strength achievable in a simulated oral environment. In order to achieve successful bonding, several factors such as the adhesiveness of the dental tissue, conditioning techniques, application modes, and the dental tissue substrate should be considered. Advances in the chemical composition and of dental adhesives have led to greater predictability and efficacy in clinical settings.

Despite the fact that in vitro studies cannot account for all aspects that lead to a material’s long-term clinical effectiveness, this study has revealed new insights into the bond strength and fluoride release of Clearfil UBQ as a universal adhesive in the treatment of primary teeth. There should be more research into its physical and mechanical qualities to determine its long-term durability and effectiveness.

## 5. Conclusions

Based on our results, we drew the following conclusions:

- Clearfil UBQ has excellent adhesive properties to primary enamel. It has an enamel shear bond strength similar to Scotchbond.
- Fluoride release was seen in Clearfil UBQ in the initial two days of the 30-day period. The amount of fluoride release was significantly less than with glass ionomer cements.
- Fuji IX emitted the most fluoride of the three materials, while Clearfil UBQ released the least fluoride. The fluoride release patterns of these materials should be considered when selecting a dental material for a specific treatment.

## Figures and Tables

**Table 1:** Mean values and standard deviation (MPa) of the shear bond strength.

Materials	Shear Bond Strength (Mean ± SD)	*p*-Value †
Clearifil Universal Bond Quick	15.82 ± 0.88	0.540
Scotchbond Universal Bond	16.25 ± 0.73	

† Independent sample *t*-test.

**Table 2:** Mean ± SD of daily fluoride release in between the three materials.

Time	Clearfil UBQ	Fuji II	Fuji IX	*p*-Value ‡
Baseline	0.11 ± 0.25 a	4.50 ± 0.97 b	19.38 ± 2.50 c	<0.001 *
Day 1	0.01 ± 0.02 a	1.24 ± 0.45 b	7.19 ± 1.22 c	<0.001 *
Day 2	0.0 ± 0.0 a	0.90 ± 0.33 b	4.31 ± 0.70 c	<0.001 *
Day 3	0.0 ± 0.0 a	0.81 ± 0.16 b	2.81 ± 0.75 c	<0.001 *
Day 4	0.0 ± 0.0 a	0.69 ± 0.13 b	2.44 ± 0.51 c	<0.001 *
Day 5	0.0 ± 0.0 a	0.65 ± 0.13 b	2.38 ± 0.50 c	<0.001 *
Day 10	0.0 ± 0.0 a	0.0 ± 0.0 a	0.98 ± 0.30 b	<0.001 *
Day 15	0.0 ± 0.0 a	0.0 ± 0.0 a	0.61 ± 0.29 b	<0.001 *
Day 20	0.0 ± 0.0 a	0.0 ± 0.0 a	0.49 ± 0.23 b	<0.001 *
Day 25	0.0 ± 0.0 a	0.0 ± 0.0 a	0.25 ± 0.21 b	<0.001 *
Day 30	0.0 ± 0.0 a	0.0 ± 0.0 a	0.04 ± 0.10 b	0.004 *

* ‡ Independent sample Kruskal-Wallis test; * *p* < 0.05 is significant; Means sharing the same alphabetical letter are not statistically different form each other at *p* < 0.05 using post hoc pairwise comparisons. Means that have different alphabetical letters are statistically different from each other at *p* < 0.05 using post hoc pairwise comparisons.

## Data Availability

Not applicable.
